# Liver Transplantation in People Living with HIV: Still an Experimental Procedure or Standard of Care?

**DOI:** 10.3390/life13101975

**Published:** 2023-09-27

**Authors:** Erica Nicola Lynch, Francesco Paolo Russo

**Affiliations:** 1 Department of Surgery, Oncology and Gastroenterology, Gastroenterology/Multivisceral Transplant Section, Padua University Hospital, 35128 Padua, Italy; ericanicola.lynch@unifi.it; 2 Department of Medical Biotechnologies, University of Siena, 53100 Siena, Italy; 3 Gastroenterology Research Unit, Department of Experimental and Clinical Biomedical Sciences “Mario Serio”, University of Florence, 50134 Florence, Italy

**Keywords:** liver transplantation, HIV, outcomes, indications, contraindications

## Abstract

Liver transplantation (LT) is the only curative treatment for various liver diseases, including acute liver failure, end-stage liver disease, and selected unresectable liver malignancies. Combination antiretroviral therapy has improved outcomes for people living with HIV (PLWH), transforming the status of acquired immune deficiency syndrome from a fatal disease to a chronic and manageable condition. These powerful antiviral therapies have not only increased the number of HIV+ enlisted patients by improving their survival but also made the use of HIV+ organs a viable option. In this review, we summarise current knowledge on the peculiarities of liver transplantation in PLWH. In particular, we focus on the indications, contraindications, specific considerations for treatment, and outcomes of LT in PLWH. Finally, we present available preliminary data on the use of HIV+ liver allografts.

## 1. Introduction

The human immunodeficiency virus (HIV) is still a very prominent disease, affecting 2.3 million (1.9 million–2.6 million) people in Western and Central Europe and North America and 38.4 million people worldwide, according to the latest UNAIDS statistics [1]. Combination antiretroviral therapies (cARTs) were introduced in the mid-1990s and have markedly improved outcomes for people living with HIV (PLWH), transforming the status of acquired immune deficiency syndrome (AIDS) from a fatal disease to a chronic, manageable condition [2]. HIV+ patients treated with cART are now more commonly subject to complications related to comorbidities, namely hepatocellular carcinoma (HCC) or end-stage liver disease (ESLD) as a consequence of hepatitis C virus (HCV) and hepatitis B virus (HBV) co-infection or alcohol-related liver disease (ALD) [3]. In fact, HIV, HCV, and HBV co-infections are common as they share similar transmission routes [4]. Furthermore, the prevalence of ALD among HIV+ patients is known to be considerably high (a pooled prevalence of 29.80% (95% CI; 24.10–35.76) in the most recent metanalysis) [5]. Finally, the ageing HIV+ population is increasingly at risk of cardiometabolic disease complications such as metabolic dysfunction-associated steatotic liver disease (MASLD). In fact, the prevalence of MASLD in PLWH ranges from 13 to 65% [6].

Liver transplantation (LT) is the only curative treatment for various liver diseases, including acute liver failure, end-stage liver disease, and selected unresectable liver malignancies [7]. Initially, HIV-positive patients were excluded from LT out of concern for the potential acceleration of AIDS progression from the use of immunosuppressive drugs and for the allocation of limited resources for patients assumed to have a poor prognosis [8]. In 2000, due to the considerable increase in the number of referrals for HIV+ candidates, the first two LT pilot trials were undertaken on HIV+ individuals at the University of California and at King’s College in London [8,9]. These pilot studies brought favourable results. In particular, they demonstrated no evidence of progression to AIDS. On the basis of the safety and efficacy reported in the pilot trials, the National Institutes of Health funded a prospective multicentre trial that confirmed excellent early graft and patient survival in PLWH [10,11]. Initially, HCV/HIV co-infection was considered a controversial indication for LT because of the poor effectiveness of interferon-based therapies and worse post-LT outcomes [12], but these issues were finally abrogated with the advent of direct-acting antivirals (DAAs) [13]. LT in HIV+ recipients is now associated with comparable allograft and survival outcomes to HIV-negative patients [11,14,15]. In light of the reassuring recent evidence, the question as to whether clinicians can now consider LT in HIV+ patients a standard procedure needs to be addressed. 

This manuscript summarises the current knowledge on the peculiarities of liver transplantation in PLWH. In the first part of the review, we discuss the main indications and contraindications for LT in HIV+ patients. In the second part, we outline the outcomes of LT in this population of recipients. Finally, we discuss the use of HIV+ liver allografts.

## 2. Indications for Liver Transplant in PLWH

Liver disease is responsible for significant morbidity and mortality among HIV+ individuals, being one of the most common causes of non-AIDS-related death for these patients [16]. HIV co-infection has proven to be a risk factor for accelerated progression to ESLD [17,18,19]. HIV is also an independent cause of liver fibrosis, as demonstrated in a study from the Center for AIDS Research Network of Integrated Clinical Systems [20]. The main mechanisms through which HIV induces liver fibrosis are oxidative stress, mitochondrial injury, immune-mediated injury, and systemic inflammation, both virus and drug-related [3,21]. Sadly, there are data showing that pretransplant cumulative survival could be significantly shorter in HIV-positive than HIV-negative transplant candidates [22]. Early mortality, mainly related to infections, appeared to be independent of ESLD or HIV infection severity, measured using the model for end-stage liver disease (MELD) score, viral load, and CD4+ cell count [22]. By the time HIV+ candidates achieve the required MELD score for organ allocation, clinical deterioration might be so severe as to exclude PLWH from LT. In addition, HIV+ patients have historically had limited access to LT due to surgeons’ reduced willingness to operate on these candidates, even when patients are still asymptomatic and when the HIV infection is well controlled with antiretroviral therapy [23]. Considering the recent evidence on the safety and efficacy of the procedure, equal access to LT should be promoted for HIV+ recipients who meet the CD4+ cell count and other eligibility criteria defined by transplant centres. Early referral might be necessary to limit pre-LT mortality related to infections, although the data are not consistent in demonstrating the lack of appropriateness of the MELD score as a predictor of pretransplantation mortality in PLWH [24]. A significant survival benefit for liver recipients with a MELD score of at least 15 has been demonstrated [11], while no specifically designed study has assessed the performance of the MELD score in predicting mortality in PLWH vs. other LT candidates; for these reasons, MELD is currently used irrespective of HIV status. As LT in PLWH will become increasingly common, further research is warranted to better understand pre-LT mortality risk factors in HIV+ candidates to ensure equal treatment possibilities to all patients with liver disease awaiting LT.

The indications for LT in PLWH have changed over the years, reflecting the advent of the effective treatment of HCV infection and the increased prevalence of different liver diseases.

### 2.1. Hepatitis C Virus

In the United States, HCV infection was the leading cause of LT in HIV-uninfected LT recipients until the introduction of DAAs in 2015 and the main indication for HIV+ patients until a few years later in 2018 [25]. This delay can be explained by the alleviation of concerns regarding the poor outcomes of HIV-HCV co-infected candidates in the pre-DAAs era, which prompted the performance of many LTs in co-infected patients only when effective anti-HCV drugs were finally available. In fact, DAAs have shown comparable effectiveness in HIV/HCV co-infected patients, as shown in a study by Chalouni et al. on 592 HIV/HCV co-infected and 2049 HCV mono-infected participants [26]. In this study, the authors found that DAA treatment led to similar sustained virological response (SVR) rates and risk of liver-related deaths and events rates in mono-infected and co-infected individuals. In contrast, HIV+ patients had a higher risk of all-cause and nonliver-related deaths and nonliver-related cancers. A recent study by Wyles et al. showed that despite HIV control, SVR did not determine a significant decrease in incident events or mortality in patients with HIV who achieved SVR, suggesting that co-infection could attenuate the beneficial impact of SVR. It should be noted that the results of this study may have been underpowered as, although improved outcomes were detected, the difference did not reach significance.

Patients co-infected with HIV/HCV can be treated with the same DAA regimens as those with HCV mono-infection. However, attention should be paid to drug interactions with cARTs [27]: sofosbuvir/velpatasvir and sofosbuvir/velpatasvir/voxilaprevir are not recommended with the inducing drugs etravirine, efavirenz, and nevirapine. Sofosbuvir/velpatasvir/voxilaprevir should not be administered with the protease inhibitors lopinavir/ritonavir and atazanavir/ritonavir; glecaprevir/pibrentasvir is contraindicated with atazanavir-containing regimens and is not recommended with other HIV protease inhibitors. In addition, the coadministration of efavirenz, etravirine, and nevirapine is not recommended as they can reduce exposure to glecaprevir/pibrentasvir. There is some evidence that administering potentially interacting drugs does not affect the effectiveness of DAAs, but this can now more easily be avoided as noninteracting antiretroviral drugs are increasingly available [28].

### 2.2. Hepatitis B Virus

According to the most recent data from the World Health Organization (WHO), about 1% of people living with HBV infection (2.7 million people) are co-infected with HIV [29]. Conversely, of all patients infected with HBV, 7.4% also have HIV [29]. HIV/HBV co-infected patients can be effectively treated with tenofovir, which is included in first-line cART for HIV infection and is also active against HBV [30]. The transplantation of HIV/HBV patients leads to excellent outcomes in terms of patient and graft survival and equally low rates of HBV reinfection as HBV mono-infected patients [14]. 

The current guidelines [31] recommend HBV immunoglobulin suspension in PLWH, although the intermittent detection of low-level viremia and higher rates of lamivudine resistance related to earlier exposure to the drug have been reported in HIV+ LT recipients [12].

### 2.3. MASLD

Metabolic-dysfunction-associated steatotic liver disease, as newly redefined by the multisociety Delphi consensus statement in 2023, affects 25% of adults in the general population and is a disease spectrum ranging from steatosis to ESLD [32,33]. Although HIV per se does not appear to be associated with an increased MASLD risk [34,35,36,37], a higher prevalence of metabolic-dysfunction-associated steatohepatitis (MASH) and hepatic fibrosis has been observed in HIV vs. primary MASLD [38]. A prevalence of MASH between 20% and 63% and of MASH with fibrosis of 14% to 63% have been reported in HIV+ patients [38,39,40]. The strongest risk factors for MASLD in HIV+ patients do not differ from uninfected individuals and include obesity, diabetes, hypertension, and dyslipidaemia [41]. In a small exploratory study by Mohammed et al. on 26 HIV-positive and 25 HIV-negative subjects with biopsy-proven MAFLD, HIV-positive patients had a lower body mass index (BMI) and were more physically active compared with HIV-negative individuals. This could suggest that in HIV+ patients, HIV infection and treatment could both participate in determining MASLD, along with more classical risk factors [42]. 

It is known that cART has deleterious metabolic effects, as it disrupts glucose control, lipid metabolism, and body fat distribution (e.g., lipohypertrophy and lipoatrophy) [43,44]. More modern cART agents have reduced the prevalence of severe lipoatrophy, but lipohypertrophy and the underlying metabolic derangements persist [45,46]. Cumulative exposure to integrase strand transfer inhibitors (INSTIs) was found to be an independent predictor of MASLD in a HIV+ patient cohort from China [47]. The use of tenofovir alafenamide (TAF) could also be implied in steatosis progression and the worsening of serum lipid levels [48,49]. HIV+ patients have a greater degree of insulin resistance than HIV-uninfected individuals. The altered glucose metabolism has been found to be associated with decreased levels of leptin and higher levels of adipokines, including increased adiponectin and soluble tumour necrosis factor receptor 1 (sTNFR1) [50]. HIV infection also causes increased levels of highly unsaturated long-chain triglycerides [51]. In the future, MASLD can be expected to become one of the main indications for LT in PLWH. 

### 2.4. Alcoholic Hepatitis

In 2019, alcohol consumption was the major risk factor for the attributable burden of disease among 25- to 49-year-olds, accounting for 2.07 million deaths of males and 374,000 deaths of females, globally [52]. In a meta-analysis by Park et al. evaluating the prevalence of modifiable cancer risk factors among PLWH in high-income countries based on data collected during 2000–2013, hazardous alcohol consumption estimated prevalence was 24%, compared with 5–15% uninfected individuals, depending on the definition of hazardous alcohol use [53]. If PLWH were directly compared to demographically similar uninfected individuals from the United States, no significant differences were detected [53]. In a systematic review and meta-analysis specifically assessing the prevalence of AUD in PLWH, which included 25 studies with 25,154 participants across developed and developing countries, the pooled prevalence estimate of AUD among PLWH was found to be 29.80% (95% CI; 24.10–35.76) [5]. Males consumed more alcohol than women (26.9% vs. 13.37%), and the pooled prevalence of AUD was higher in developed (42.09%) vs. developing countries (24.52%) [5]. Increased daily alcohol use (>50 g) was unsurprisingly correlated with a higher risk of liver fibrosis (pooled OR = 3.10, 95% CI: 2.02–4.73, *p* < 0.05) among PLWH [54]. In a study conducted by the Comprehensive Alcohol Research Center (CARC) in Louisiana on 353 HIV+ patients actively on treatment, the prevalence of intermediate liver fibrosis ranged from 41.1 to 64.3% in HIV/HCV co-infected patients and 8.8–45.8% among HIV mono-infected individuals [55].

## 3. Specific Eligibility Criteria and Contraindications for Liver Transplant in HIV+ Recipients

HIV-specific eligibility criteria for LT have varied in light of reassuring evidence supporting the safety and efficacy of solid organ transplantation in PLWH. In the HIV-TR study by Roland et al., the eligibility criteria for LT were the CD4+ T lymphocyte count > 100 cells/mL and any HIV-1 RNA count as long as a fully suppressive antiretroviral regimen was likely to be tolerated post-transplant [11]. In this study, the severity of HIV infection, measured using the CD4+ cell count at baseline and transplantation, CD4+ T-cell count nadir, and pre-LT opportunistic infections, did not affect graft and patient survival, validating the HIV-related eligibility criteria. The only significant predictor of loss of virologic control was the discontinuation of ART. These results highlight the importance of the current immune status and degree of viral control rather than the HIV clinical history in pretransplant evaluation. 

**Figure 1:**
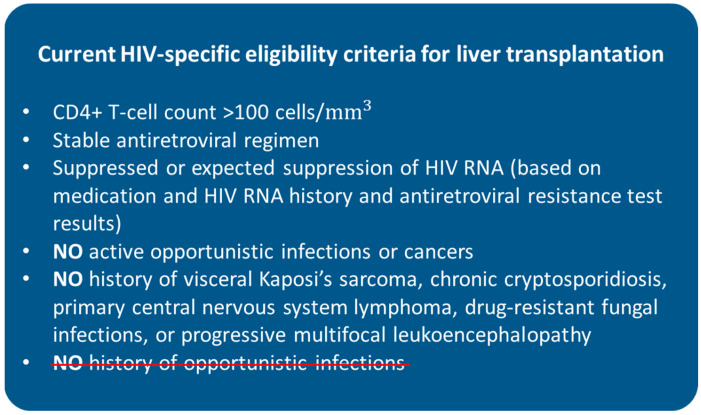
Current HIV-specific eligibility criteria for liver transplantation. HIV = human immunodeficiency virus. History of opportunistic infections used to be a contraindication for liver transplantation in HIV-positive patients.

The current selection criteria can vary among different transplant centres, though they generally include a minimum CD4+ T-cell count of 100 cells/mm^3^, stable antiretroviral regimen (when feasible), suppressed or expected suppression of HIV RNA (based on medication and HIV RNA history and antiretroviral resistance test results), no active opportunistic infections or cancers, and no history of visceral Kaposi’s sarcoma, chronic cryptosporidiosis, primary central nervous system lymphoma, drug-resistant fungal infections, or progressive multifocal leukoencephalopathy [8,56,57]. HIV RNA levels are commonly required to be undetectable within 3–4 months from LT, although candidates who are not able to tolerate cART because of drug-associated hepatotoxicity are considered eligible for transplantation if viral suppression can be confidently predicted to occur after LT [58,59]. Having a history of opportunistic infections used to be an absolute contraindication but has been removed from the criteria as it has been shown it has no impact on outcomes, as in the above-mentioned HIV-TR study [11,60]. The present suggested eligibility criteria are summarised in Figure 1.

The last and only study assessing the willingness of LT surgeons to provide grafts to PLWH dates back to 2005 [23]. In this study, only one third of LT surgeons considered PLWH appropriate LT candidates: survival estimates alone did not explain the surgeons’ choices nor did the fear of personal harm. The majority of surgeons believed HIV to be a contraindication for LT probably due to the residual influence from their poor experiences in transplanting such patients before the advent of cART. Surgeons who had previously been exposed to HIV were more reluctant to operate on PLWH. As the clinical scenario of HIV infection has deeply changed since 2005, surgeons’ willingness to perform LT in PLWH should be reassessed.

## 4. Outcomes of Liver Transplant in PLWH

### 4.1. Graft and Patient Survival

Since LT in HIV+ patients was first performed in 2000, there still is a scarcity of data on the long-term (>10 years) outcomes in this specific population. The first long-term analysis in HIV+ LT used data from the Scientific Registry of Transplant Recipients and compared 180 HIV+ LT recipients with matched HIV-negative controls over 10 years of follow-up after LT. The authors found that HIV+ LT recipients had a 1.68-fold increased risk of death compared to HIV-negative patients and that these differences were independent of HCV status. However, when restricting the transplant era to >2008, the risk of death was similar between HIV+ mono-infected and uninfected LT recipients. INSTIs have become the recommended ART regimen in the transplant setting since their introduction in 2008, as they do not interact with standard immunosuppressants. INSTIs could be at least partly responsible for the improved outcomes in the modern transplant era. On the other hand, the mortality of HIV/HCV co-infected LT recipients was higher than mono-infected patients in all transplant eras, but the follow-up period of this study ended in 2011 before DAAs were introduced [61]. 

More recently, Zarinsefat at al. presented the results of the HIV+ LT programme of the University of California, San Francisco (UCSF) [62]. Since UCSF pioneered the procedure, the follow-up period is 20 years. A total of 80 HIV+ LT patients were included and appropriately matched with a HIV-negative cohort. Patient survival was not significantly different between the two groups: 75.7% (95% CI, 71.8–79.8%) for HIV-negative patients and 70.0% (95% CI, 60.6–80.8%) for HIV-positive LT at 15 years post-LT, with a stratified log-rank test that was not statistically significant (*p* = 0.12). Survival markedly increased in the DAA era, with only 59.5% of patients surviving at 5 years post-transplant pre-DAAs vs. slightly under 90% after 2014. The advent of DAAs has, therefore, abrogated the poor outcomes that were previously seen in HIV/HCV co-infected LT recipients, although long-term (>5 years) data are lacking due to the recent introduction of these drugs.

### 4.2. HIV-Specific Outcomes

#### 4.2.1. Infections

In a large study on the United Network for Organ Sharing (UNOS) data from 64,977 patients who underwent liver transplantation between 2002 and 2016, infections were the prevalent cause of early mortality between 30 and 180 days after LT (30-day, *n* = 12, 36.4%; 90-day, *n* = 5, 55.6%; 120-day, *n* = 5, 50.3%; 180-day, *n* = 7, 46.7%) [63]. Unfortunately, HIV infection was not included in the assessment of risk factors. Available data from the pre-DAA era suggest that HIV+ recipients are more commonly subject to bacterial infections typically found in HIV-negative recipients, especially HIV/HCV co-infected patients and those treated with antithymoglobulins [10,59]. 

The reported incidence of infections after LT in PLWH differs among studies available in the literature [64]. In the pre-DAA era, severe infections greatly affected the outcomes of solid organ transplant in HIV/HCV co-infected LT recipients [10,65]. In a prospective study by Moreno et al. on 84 consecutive HIV/HCV co-infected patients with a median follow-up of 2.6 years, 64% developed at least one infection, and 11% developed opportunistic infections with a 44% mortality rate [65]. In a small study on 27 HIV+ LT recipients vs. 27 HIV- LT recipients with a median follow-up of 26/27 months, none died because of infections [66]. A total of four HIV+ patients developed opportunistic infections (oesophageal candidiasis and *Pneumocystis jirovecii* pneumonia after prophylaxis discontinuation), whereas there were none in the control group [66]. In fact, when prophylaxis is appropriately administered, AIDS-related infections are possible but uncommon after LT [67,68]. Miro et al. reported comparable infection rates but higher tuberculosis and fungal infections in PLWH after LT [64]. Although the available data can be considered reassuring, larger multicentre studies should be conducted to increase the numerosity of the included population of HIV+ LT recipients and investigate current infection rates and infection-related mortality in the DAA era, both for bacterial and opportunistic infections. The role of induction immunosuppression with antithymoglobulins should also be assessed.

The data on coronavirus disease 19 (COVID-19) in HIV+ LT recipients are scarce and inconsistent [69], although mortality rates as high as 36% have been reported. COVID-19 in nontransplanted PLWH appeared to have similar outcomes to those of HIV-negative patients [70,71].

#### 4.2.2. Malignancies

Several studies highlight a high risk of non-AIDS-defining cancers in HIV populations due to chronic inflammation and immune system dysregulation related to HIV infection [72,73,74] but also linked to high-risk behaviours, such as tobacco use and alcohol consumption [75]. Overall, HIV+ LT recipients do not have an increased risk of cancer compared to uninfected controls [76]. Malignancies associated with human papillomavirus have been reported more frequently in HIV+ solid organ recipients [10,77], but the most common cancers after LT in HIV+ patients are Kaposi’s sarcoma (standardized incidence ratio—SIR = 451 in HIV-positives, 125 in transplants) and non-Hodgkin lymphoma (SIR = 62 and 11.1, respectively) [76]. Particular attention should be given to identifying additional risk factors for cancer development and oncological surveillance in this particular population. 

HCC accounts for nearly half of liver-related mortality in PLWH [78]. HCC seems to have a more aggressive course in HIV+ patients, possibly related to a more profound T cell dysfunction in PLWH compared with controls [79]. Fortunately, the outcomes of LT for HCC do not appear to be affected by HIV infection [57,80,81,82,83].

#### 4.2.3. Graft Rejection

Even though HIV infection leads to immunodepression, it has been noted that acute rejection (AR) rates are 2/3-times higher in HIV+ kidney [84,85], liver [62] and heart [86] recipients. Early (<6 months after LT) rather than late rejection episodes seem to be more frequent [85,86].

The possible explanations for higher rates of AR in HIV+ patients could be the drug interaction between cART and immunosuppressive drugs, especially involving protease inhibitors [77], the increased responsiveness of T-cells and the nonspecific enhancement of alloimmune reaction in PLWH [87], and more classical risk factors such as previous allosensitisation [10]. There are no data that establish an optimal time length for which PLWH should be on cART before undergoing LT. However, longer periods of viral control prior to LT may decrease immune activation, potentially reducing the risk of AR, as shown in a study on kidney transplant recipients [88]. In this study by Husson et al., the rates of AR were not influenced by induction immunosuppression but were 2.48 times lower in patients who had obtained HIV suppression at least two years before LT [88]. 

## 5. Immunosuppression and HIV Antiviral Treatment

As indicated in the current guidelines, PLWH generally receive the same immunosuppressive regimens as HIV-negative LT recipients [59]. cART is usually administered until liver transplantation in order to suppress plasma HIV RNA and is resumed once the patient is stable and oral intake can be reintroduced [59]. 

Despite the potential antiviral activity against HIV of cyclosporin, tacrolimus should be the preferred immunosuppressive drug in HIV+ recipients as it reduces rejection rates as in HIV-uninfected patients [89]. Long-term low-dose corticoid treatment might be advisable to further reduce the risk of AR [59]. The mTOR inhibitor sirolimus has been shown to enhance the antiviral activity of cART and is less nephrotoxic than tacrolimus, but its use could determine higher AR rates [59].

Immunosuppression induction with thymoglobulin in PLWH has been a matter of debate, as there is conflicting data both suggesting the association of its use with decreased patient and graft survival in kidney transplant recipients but also with decreased rejection rates in LT [10,11,90]. Until more solid data are available, thymoglobulin induction cannot be recommended in PLWH [59]. 

Due to drug–drug interactions, the preferred cART for HIV+ LT recipients should not contain a protease inhibitor or pharmacokinetic enhancer such as cobicistat [59]. If this is unavoidable, significant dose adjustments to immunosuppressive drugs should be performed.

## 6. Possible Use of Organs from HIV+ Donors 

Advances in transplant medicine have markedly improved the outcomes of liver transplantation [91]. As a result of this success, coupled with the receding numbers of donors after brain death, the demand has outstripped the availability of organs, leading to higher waitlist mortality rates [92,93]. In 2002, the MELD system was introduced to minimise waitlist mortality by prioritising the sickest recipients [94]. Despite the increasing acuity of patients undergoing LT, in the post-MELD era, better overall graft and patient survival were achieved, with significant reductions in 30-day mortality rates [95]. Undoubtedly, many factors have contributed to the improvement in post-transplant outcomes, such as better patient selection, perioperative management, immunosuppression, and technical advancements [96]. 

To further reduce waitlist mortality, organ shortage has been addressed by expanding the criteria for donor selection, prompting clinicians to accept allografts that were previously deemed unsuitable for transplantation [97]. According to the Eurotransplant annual reports, the donor age is progressively increasing [98], and in 2012, over 50% of liver allografts within the Eurotransplant region were considered extended [99]. 

Owing to the increased survival of HIV+ individuals in the cARTs era, the number of HIV+ patients enlisted for LT has grown. These powerful antiviral therapies not only have increased the life expectancy of affected patients but also have made the use of HIV+ organs a viable option. The first kidney transplants from HIV+ donors were performed in South Africa and were associated with excellent outcomes, also in terms of the opportunistic infection rates [100]. No evidence of superinfection with more resistant strains was reported. Following this positive experience and in order to facilitate access to LT for PLWH, liver transplant centres in the United States have fought to change the federal laws to allow the use of organs from HIV+ donors. In 2013, the HIV Organ Policy Equity (HOPE) Act was enacted, allowing HIV D+/R+ transplantation within investigational protocols, whose criteria were defined in 2015 [101]. HIV-uninfected patients also benefit from the use of HIV+ grafts through the increased overall number of available organs, including HIV+ false positive grafts which would have been discarded and now can be used in PLWH [102]. Recipient eligibility criteria for HIV+ organs include viral suppression using cART, which should reduce the risk of superinfection with a resistant strain. HIV D+/R+ solid organ transplantation is currently under investigation in clinical trials (NCT03500315 and NCT03734393). The preliminary results are promising in terms of graft and patient survival, although a trend towards higher 1-year AR rates has been reported in the kidney transplant group, with a possible protecting influence of antithymoglobulin [103,104]. In LT, higher rates of opportunistic infections (primarily cytomegalovirus infections) and cancer were observed in patients receiving HIV+ organs [103]. Among the reported cancers, three were related to human herpesvirus 8 (HHV8) [103]. The main characteristics and data from the above-mentioned studies on the use of organs from HIV+ donors are illustrated in Table 1. With definitive data, the risks associated with the use of HIV+ organs should be weighed against the potential benefits of using HIV+ grafts. Recent studies indicate high willingness among PLWH to accept grafts from HIV+ donors [105]. 

## 7. Conclusions

**Figure 2:**
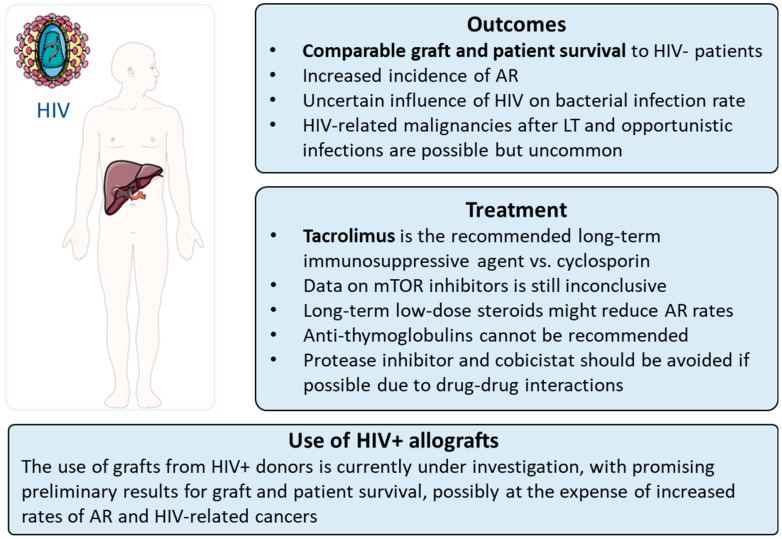
Main peculiarities of liver transplantation in HIV+ patients. HIV = human immunodeficiency virus.

Liver transplantation in people living with HIV is associated with comparable outcomes to HIV-uninfected LT recipients in terms of graft and patient survival. For this reason, LT in PLWH should be considered standard practice, although there are peculiarities of HIV+ LT recipients which should be taken into account (as summarised in Figure 2). The incidence of acute rejection appears to be increased in PLWH, while the rate of infections after LT varies among studies, with data lacking from the DAA era. Opportunistic infections and HIV-related malignancies after LT are possible but uncommon. Drug–drug interactions and a higher risk of AR should guide the choice and dose of immunosuppressive therapies. The use of grafts from HIV+ donors is currently under investigation, with promising preliminary results for graft and patient survival, possibly at the expense of increased rates of AR and HIV-related cancers.

## Figures and Tables

**Table 1:** Studies on solid organ transplantation using HIV+ donors. cART = combined antiretroviral therapy; AIDS = acquired immune deficiency syndrome; KT = kidney transplant; LT = liver transplant: HIV = human immunodeficiency virus; CNS = central nervous system; CI = confidence interval; D = donor.

Authors, Year	Study Type	Follow-Up (Years)	Organ	Donor(s) Characteristics	Recipient(s) Characteristics	Post-Transplant Outcomes
**Muller et al., 2015** [106]	Monocentre, prospective, pilot, observational study	5	Kidney	**Inclusion criteria:** Deceased, no prior cART or on first-line treatment, undetectable plasma HIV RNA viral load (<50 copies per milliliter) **Exclusion criteria:** severe sepsis, active tuberculosis, World Health Organization stage 4 HIV disease (i.e., AIDS) and abnormal renal function as assessed by the serum creatinine level, the presence of proteinuria on the urine dipstick, or a microalbumin-to-creatinine ratio >300 μg of microalbumin per milligram of creatinine	**Inclusion criteria:** cART for at least 3 months, CD4 T-cell count =/>200 per cubic millimeter, and undetectable plasma HIV RNA viral load **Exclusion criteria:** a history of any opportunistic infection that suggested a diagnosis of AIDS, a history of drug-sensitive tuberculosis without completion of a minimum of 6 months of treatment	**D HIV+ patient survival:** 84% (95% CI, 62 to 94) at 1 year and 3 years and 74% (95% CI, 45 to 89) at 5 years **D HIV+ graft survival:** 93% (95% CI, 74 to 98) at 1 year, 84% (95% CI, 55 to 95) at 3 and 5 years **D HIV− patient survival:** 91% (95% CI, 63 to 92) at 1 year and 85% (95% CI, 56 to 94) at 5 years **D HIV− graft survival:** 88% (95% CI, 47 to 91) at 1 year and 75% (95% CI, 43 to 88) at 5 years
**Durand et al., 2021** [103]	Multicentre, prospective, pilot, observational study	1.7	Kidney	**Inclusion criteria:** no specific criteria for donor HIV RNA or CD4 count, being effectively on cART **Exclusion criteria:** no active opportunistic infection or cancer	**Inclusion criteria:** ≥18 years of age, CD4 ≥ 200 cells/μL within 16 weeks of KT, on cART with HIV RNA < 50 copies/mL **Exclusion criteria:** active opportunistic infection or history of progressive multifocal leukoencephalopathy or CNS lymphoma	**D HIV+ patient survival:** 100% with a median follow-up of 1.4 years **D HIV+ graft survival:** 91% (95% CI 67–98%) at 1 year **D HIV− patient survival:** 100% with a median follow-up of 1.8 years **D HIV− graft survival:** 92% (95% CI 80–97%) at 1 year
**Durand et al., 2022** [104]	Multicentre, prospective, pilot, observational study	1.9	Liver	**Inclusion criteria:** no specific criteria for donor HIV RNA or CD4 count, being effectively on cART **Exclusion criteria:** no active opportunistic infection or cancer	**Inclusion criteria:** ≥18 years of age, CD4 ≥ 100 cells/μL within 16 weeks of LT, on cART with HIV RNA < 50 copies/mL, liver-kidney transplants were permitted **Exclusion criteria:** active opportunistic infection or history of progressive multifocal leukoencephalopathy or CNS lymphoma	**D HIV+ patient survival:** 83.3% at 1 year **D HIV+ graft survival:** 96% at 1 year **D HIV− patient survival:** 100% at 1 year **D HIV− graft survival:** 100% at 1 year

## Data Availability

No new data were created or analysed in this study. Data sharing is not applicable to this article.
